# Oxidation of Hydrocarbons on the Surface of Tin Dioxide Chemical Sensors

**DOI:** 10.3390/s110404425

**Published:** 2011-04-15

**Authors:** Helena Teterycz, Patryk Halek, Kamil Wiśniewski, Grzegorz Halek, Tomasz Koźlecki, Izabela Polowczyk

**Affiliations:** 1 Faculty of Microsystem Electronics and Photonics, Wrocław University of Technology, 50-372 Wrocław, Poland; E-Mails: patryk.halek@pwr.wroc.pl (P.H.); kamil.wisniewski@pwr.wroc.pl (K.W.); grzegorz.halek@pwr.wroc.pl (G.H.); 2 Faculty of Chemistry, Wrocław University of Technology, 50-373 Wrocław, Poland; E-Mails: tomasz.kozlecki@pwr.wroc.pl (T.K.); izabela.polowczyk@pwr.wroc.pl (I.P.)

**Keywords:** gas sensor, molecular structure, alcohol, oxidation reaction, Lewis acidity

## Abstract

The paper presents the results of our investigation on the effect of the molecular structure of organic vapors on the characteristics of resistive chemical gas sensors. The sensors were based on tin dioxide and prepared by means of thick film technology. The electrical and catalytic examinations showed that the abstraction of two hydrogen atoms from the organic molecule and formation of a water in result of reaction with a chemisorbed oxygen ion, determine the rate of oxidation reactions, and thus the sensor performance. The rate of the process depends on the order of carbon atoms and Lewis acidity of the molecule. Therefore, any modification of the surface centers of a sensor material, modifies not only the sensor sensitivity, but also its selectivity.

## 1. Introduction

It is commonly known that resistive semiconductor chemical sensors are made of common ceramic resistors, which receive different chemical signals and transform them into readable responses. The reception and transformation of the chemical signals arises as a result of multi-stage heterogenic and catalytic chemical reactions taking place at the surface of gas-sensitive materials. The reactions are accompanied by the electric charge transfer from the sensitive material to the gas atmosphere and *vice versa*. The kinetics of the processes, which take place in the receptor part, determine the sensor parameters, like sensitivity, selectivity, working temperature and response time. It is commonly accepted that the parameters depend on the sensor material and its microstructure, developed during the technological fabrication process [1–9]. However, for the reactions in the receptor part of the sensor, not only the surface layer takes part in the process, but also gas molecules with an appropriate structure participate equally. For this reason, the essential sensors parameters like sensitivity, selectivity, response time and optimal working temperature depend on several factors:

▪ efficiency of receptor part,
▪ efficiency of actuator part,
▪ properties of sensor design.

Efficiencies of both receptor and actuator parts depend on:

▪ electronic structure of sensing material,
▪ density of surface states,
▪ amount and quality of absorption centers,
▪ microstructure of sensing material,
▪ catalytic activity,
▪ kind of detected gas.

In the scientific literature there are many works devoted to the effect of the chemical structure of sensing material on the first four factors mentioned above [3,4,10–16]. There are, however, almost no works devoted to the assessment of the effect of shape and size of the detected molecules on the sensor characteristics [17–19]. For efficient modification of the parameters of the resistive sensor, a comprehensive analysis of phenomena occurring during the analysis of chemical substances is necessary. It is particularly important to improve the sensor selectivity, since it depends on the efficiency of receptor part, but its transducer part does not show any chemical selectivity. For this reason, the aim of the work was to assess the effect of molecule structure of organic vapors on the performance of resistive sensors.

## 2. Experimental

Tin dioxide was obtained by means of the sol-gel technique. A small amount of a mixture of anhydrous tin chloride (IV) and glacial acetic acid was added to a large volume of distilled water at 80 °C. A fine sediment of hydrated tin dioxide was precipitated and rinsed with distilled water until negative chloride ions test results were obtained. Then it was washed with a small amount of 2-propanol and dried at 50 °C. After annealing of the powder at 600 °C for 30 min, a paste with appropriate rheological properties was made. The sensors were fabricated by printing a gas-sensitive material on alumina substrates. The electrodes were made of gold paste. Gas-sensitive material was printed twice and the process was followed by firing at a temperature of 850 °C for 2 h.

The crystallographic structure of the SnO_2_ powders and gas sensitive layers was determined using a Philips Materials Research Diffractometer (MRD) X-ray diffractometer with CuK_α_ radiation. The Θ/2Θ scan, typical for powder materials, was used. The average grain sizes and distributions were determined on the full width at half maximum (FWHM) of the peaks and diffractometric profile analysis. AWPX software was used for this purpose. The microstructure of gas-sensitive material was observed with JSM 5800 LV scanning electron microscope (Jeol), equipped with an ISIS 3000 X-ray microanalysis system (Oxford).

Electrical investigations of resistive gas sensors were made by thermo stimulated conductance method, using a SI 1287 electrochemical interface (Solartron). The temperature of the sensor heater was varied between 150 °C to 750 °C at a constant heating rate of 2 °C/s and the corresponding change in the electric current flowing through the sensor at a constant bias voltage was registered. The studies were carried out in an ambient atmosphere at 30% relative humidity, in the presence of vapors of various aliphatic organic compounds: ethanol, 2-propanol, propanone (acetone), 2-methyl-2-propanol and *n*-butanol. The compounds were selected on the basis of different factors: carbon chain length (2–4), linear or branched structure and different chemical functionality (alcohols and ketone).

The catalytic activity of tin dioxide was evaluated measuring the oxidation of *n*-butanol. The powder of SnO_2_ was pellized at 80 MPa, crushed and sieved to obtain homogeneous grains with diameters between 0.6 and 1.2 mm. The reaction was accomplished in a vertical, quartz, flow type reactor—Thermolyne Tube Furnace F21100, filled with 3 cm^3^ of grains. The reaction was carried out continuously in the gas phase, at atmospheric pressure and the temperature range of 250–450 °C fixed to a few specified levels. No carrier gas was used. The measurements of the yield and selectivity were performed after the reaching of stationary equilibrium of the reaction. The products of the reaction were analyzed using a HP 6890 gas chromatograph, coupled with HP MSP 59 mass spectrometer. Both yield and selectivity have been determined.

## 3. Results and Discussion

**Figure 1.**
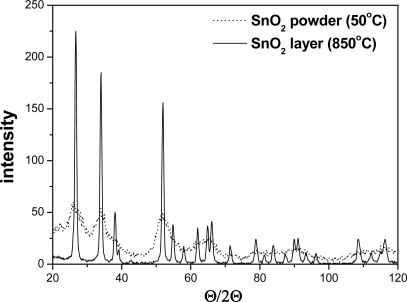
X-ray diffractogram of the powder and the thick film SnO_2_.

The X-ray investigation of the powder and layer of the pure tin dioxide revealed its typical crystallographic structure, *i.e.*, the rutile type (Figure 1).

The mean particle diameter was calculated from the XRD pattern according to the line width of the plane refraction peak using the following Debye-Scherrer equation:

(1) D=K⋅λβ12⋅cos Θ

where K is the shape factor, λ is the X-ray wavelength, β is full width half maximum (FWHM) of the diffraction peak and θ is the Bragg diffraction angle of the of XRD peak in degrees. The average crystallite size of the tin dioxide crystals calculated from peak width is about 32 nm.

**Figure 2.**
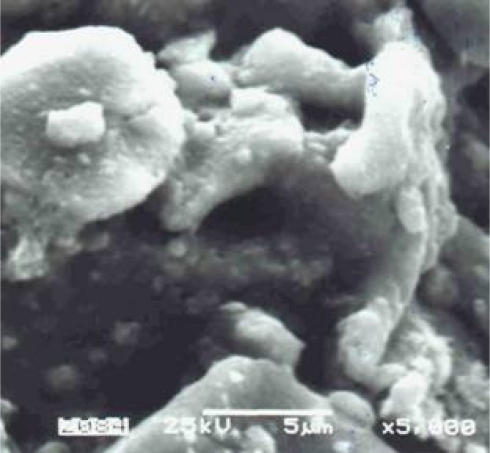
SEM micrograph of gas sensing layer of tin dioxide.

SEM micrographs showed that the gas-sensing layers were made of monocrystallites arranged in a very porous structure (Figure 2).

**Figure 3.**
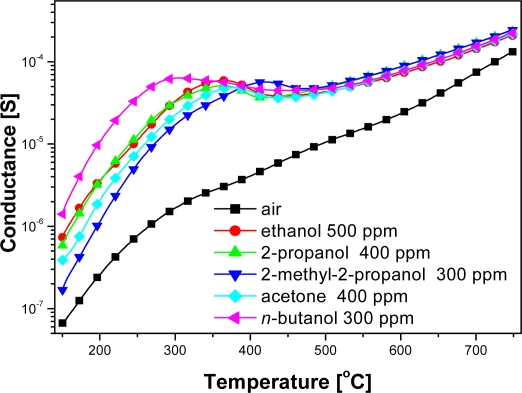
Temperature dependence of conductance at the atmosphere containing various organic compounds.

Electrical measurements showed that the conductance of examined sensors in pure air was significantly lower than in the ambient containing organic compounds vapors over the whole temperature range (Figure 3). At the temperatures below 500 °C the changes in the sensor conductance was highly dependent on the structure of the organic compound. The peak value of the sensors conductance in ethanol (a straight-chain 2-carbon alcohol) did not differ markedly from the sensors conductance in the ambient containing 2-propanol (branched-chain 3-carbon alcohol), in spite of the fact that according to the Equations (1) and (2) in the reaction of 2-propyl alcohol with chemisorbed oxygen much more electrons are injected to conduction band than in ethanol reaction (Figure 3):

(2) $$C_{2}H_{5}{\text{OH}+6O}^{-}\to {2\text{CO}}_{2}+{3H}_{2}O+{6e}^{-}$$

(3) $${\text{CH}}_{3}{\text{CH(OH)CH}}_{3}{+\text{ 9O}}^{-}\to {3\text{CO}}_{2}{+4H}_{2}O+{9e}^{-}$$

(4) $${\text{CH}}_{3}{\text{CH}}_{2}{\text{CH}}_{2}{\text{CH}}_{2}\text{OH}+{12O}^{-}\to {4\text{CO}}_{2}+{5H}_{2}O+12e^{-}$$

The highest conductance had the sensors in the atmosphere of *n*-butyl—a four-carbon alcohol—however, in the atmosphere of 2-methyl-2-propanol, which also contains four carbons atoms in its molecule, the conductance was the lowest (Figure 3). Therefore conductance changes of chemical gas sensors on organic compounds couldn't be explained by commonly accepted theory, that in processes of detection, organic gases are combusted as indicated in Equations (2–4) [11].

**Figure 4.**
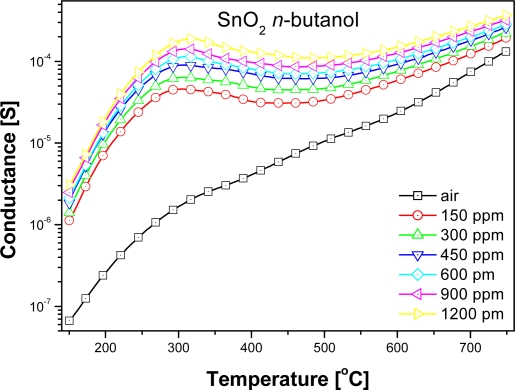
Temperature dependence of sensor conductance in the atmospheres with different concentration of *n*-butanol.

At high temperatures, above 500 °C, the values of the sensors conductance were almost unrelated to the nature of alcohol (Figure 3), but depended on the concentration of sensed compound (Figure 4).

**Figure 5.**
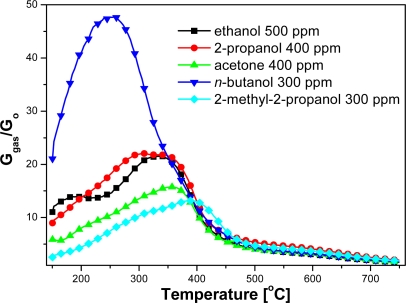
Temperature dependence of sensors sensitivity in the presence of different organic compounds.

**Figure 6.**
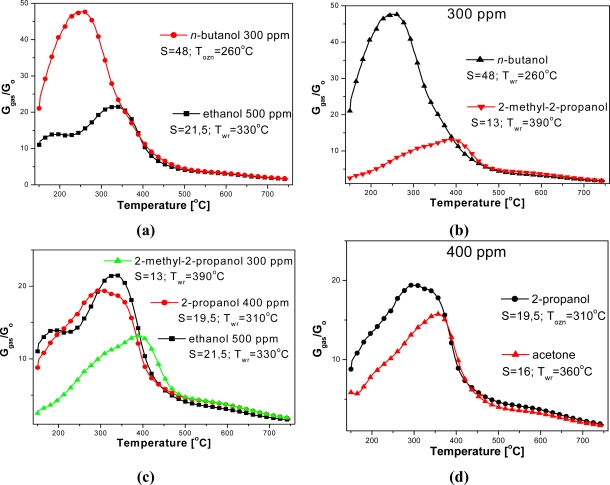
Temperature dependence of sensors sensitivity on **(a)** the number of carbon atoms in the molecule, **(b)** the molecule shape, **(c)** order of alcohol, **(d)** kind of functional group.

Based on electrical measurements of the sensors, carried out in the gas atmosphere with different compositions, the dependence of sensors sensitivity on temperature in a wide temperature range has been estimated (Figures 5 and 6). Organic compound concentrations in measurements were selected to obtain comparable numbers of carbon atoms in the test atmospheres.

The sensor sensitivity *S* has been defined as a ratio of the conductance in the atmosphere containing the vapors of assayed compound (*G*_gas_) to the conductance in the ambient atmosphere (*G*_o_):

(5) $$S=\frac{G_{\mathrm{gas}}}{G_{o}}$$

Based on temperature dependence of sensitivity on the kind and concentration of assayed compound, optimum sensing temperatures of tested alcohols have been estimated (Table 1). The optimum temperature of sensing for an assumed concentration of tested compounds was related to the length of carbon chain, molecule shape and kind of functional group (Table 1). The tested sensors revealed the lowest sensing temperature, equal to 260 °C, in the presence of a primary alcohol—*n*-butanol, whose molecule with a straight linear chain consists of four carbon atoms (Table 1). The sensors displayed a higher sensing temperature, up to 380 °C, in the atmosphere containing ethanol or 2-propanol alcohol vapors. Ethanol is a primary alcohol and its molecule contains two carbon atoms, whereas 2-propanol is a secondary alcohol with three carbon atoms in the molecule. The highest working temperature, equal to 390 °C, was observed at the presence of 2-methyl-2-propanol vapors, a ternary alcohol, containing, just like *n*-butanol, 4 carbon atoms in the molecule.

The highest sensitivity (73.4) was achieved in the atmosphere of *n*-butanol, being three times higher than in the presence of ethanol (23.8) [Table 1; Figure 6(a)]. But in the presence of the same amount of 2-methyl-2-propanol the sensitivity of the sensors was the lowest, reaching barely 13.2 [Figure 6(b)]. Sensitivity decreases significantly from primary to secondary, then tertiary alcohols [Figure 6(c)]. Furthermore, changing the functionality, from alcohol to ketone, also reduces the selectivity [Figure 6(d)].

**Figure 7.**
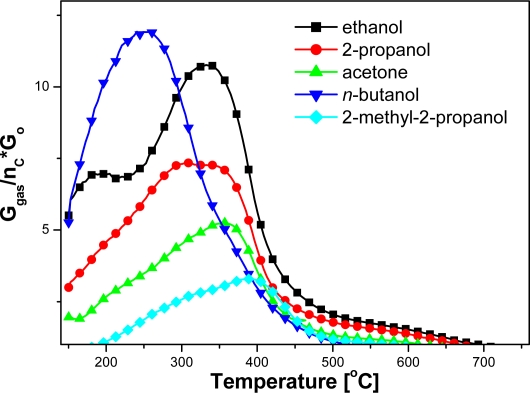
Temperature dependence of sensitivity value divided by the number of carbon atoms in the molecule.

The effect of molecular structure and the number of carbon atoms in the molecule on the sensitivity and working temperature is all the more apparent, when the sensitivity value is divided by the number of carbon atoms (Figure 7). It can be seen that the relative reactivity of the both primary alcohols is very close and decreases with an increase of alcohol order.

It is commonly accepted that the value of the output signal of a resistive sensor (conductance) at the working temperature (the temperature at which a sensor exhibits the highest sensitivity), *versus* partial pressure of tested gas is described by the following formula:

(6) $$G_{\mathrm{gas}}=A\cdot p_{\mathrm{gas}}^{n}$$

where *A* is the constant and *n* is the experimentally determined exponent. The value of *n* depends on the kind of gas-sensitive material, the employed modifying additive and the kinetics of reaction of the assayed gas with oxygen chemisorbed on the surface of gas sensitive material. Since in the current work, the sensors with a gas sensitive layer made of pure SnO_2_ have been employed and the assayed gases made the same homologous series of organic compounds it can be inferred that the differences in the value of n exponent characterize the effect of molecule structure on the value of the exponent.

Combining Equations (5) and (6), after dividing the value of sensitivity by the number of carbon atoms in the molecule, the following relationship has been obtained:

(7) $$S_{C}=\frac{S}{n_{C}}=\frac{A\cdot p_{\mathrm{gas}}^{n}}{n_{C}\cdot G_{o}}=A\prime \cdot p_{\mathrm{gas}}^{n}$$

where *A’* is the constant, *S*_C_ is the modified sensitivity. Finding a logarithm of the above relationship, the following equation has been obtained:

(8) $$\text{lg}\;S_{C}=\text{lg}\frac{S}{n_{C}}=\text{lg}\;A\prime +n\;\text{lg}\;p_{\mathrm{gas}}$$

**Figure 8.**
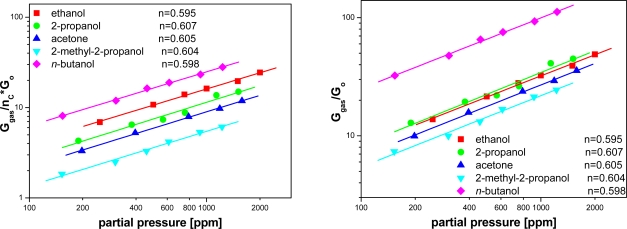
Modified sensitivity dependence *versus* partial pressure of alcohols.

Drawing the dependence of the modified sensitivity *versus* gas pressure on a log-log scale, the value of *n* has been estimated (Figure 8). The estimated values of *n* exponent are the same within the error margin and did not depend on the differences in the structures of the assayed organic compound molecules. Since *n* does not depend on the composition of examined atmosphere we can infer that the reactions occurring at the surface of SnO_2_, with the contribution of the organic compounds, have a multi-stage character but follow a similar scheme.

In general, the rate of oxidation reaction ν of the tested substances at the surface of sensing material, can be described by the following equation:

(9) $$v=k\cdot {\left[O^{-}\right]}^{a}\cdot p_{\mathrm{oc}}^{n}$$

where ***k*** is the reaction rate, [*O*^−^] is concentration of oxygen ions chemisorbed at the surface of gas-sensing material, ***p****_oc_* is the partial pressure of assayed organic compound, and ***a***, ***n*** are the power exponents.

According to [20] in the temperature range, where the highest value of the sensors sensitivity is observed in the presence of the examined organic compounds, *O*^−^ ion is the dominant form of the oxygen chemisorbed at SnO_2_ surface. Since the partial pressure of oxygen did not change during the experiment, one can claim that the oxidation reaction rate of the examined organic compounds at a given temperature depends only on the partial pressure of analyzed compound. For this reason, the exponent ***n*** from Equation (9) is the same as the one in Equation (6).

The performed measurements showed that ***n*** does not depend on the kind of assayed organic compound. This means that the oxidation reaction rates of the compounds at the surface of tin dioxide are affected to the same degree by the increase in concentration of each of the assayed compounds. Due to this fact it can be stated that the reactions occurring with the contribution of examined organic compounds on SnO_2_ surface are multi-stage, but follow the same scheme. Furthermore, it means that the first stage of the reaction, depending on the kinetics of interactions of a molecule of organic compound with the sensor surface, determines the rate of the whole oxidation process and thus the variation of the sensor conductance.

The organic compounds analyzed during electric measurements differed by:

✓ number of carbon atoms in the molecule,
✓ arrangement of carbon atoms in the molecule,
✓ location of hydroxyl group,
✓ kind of functional group.

The differences in structure of assayed compounds caused that they differed additionally by:

✓ electric charge distribution in the molecule,
✓ dipole moment,
✓ ease of dehydrogenation and dehydration,
✓ ease of oxidation.

**Figure 9.**
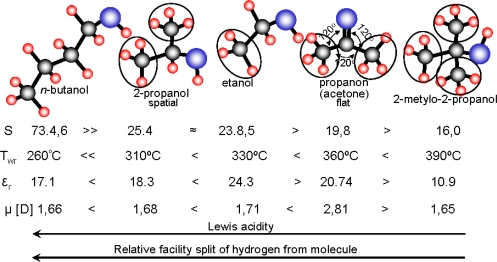
Shape of examined organic compounds, sensitivity and working temperature of sensors, dipole moment and relative electric permittivity as well as the changes in Lewis acidity and relative ease of removing of hydrogen atom from molecule .

Analyzing the sensitivity and working temperature dependence on physical properties of examined compounds like: number of carbon atoms in the molecule, dipole moment and electric permittivity, characterizing electric charge distribution in the molecule, the authors have not observed any connection between the parameters (Table 1, Figure 9). However, an additional analysis of the properties let us to link the sensitivity and working temperature of examined sensors with acidity and ease of hydrogen atoms removing from alkyl groups (Figure 9).

For aliphatic alcohols, the Lewis acidity decreases with the increase of the number of alkyl groups whereas the ease to abstract the hydrogen from the alkyl group depends on the order of carbon atoms [21,22]. From this reason, analyzing possible oxidation reaction pathways of examined compounds at the surface of tin dioxide, the authors reasoned that the rate-determining step of the whole process is the abstraction of two hydrogen atoms from the molecule, followed by the formation of water molecule as a result of reaction of the hydrogen atoms with a chemisorbed oxygen ion (10):
(10)
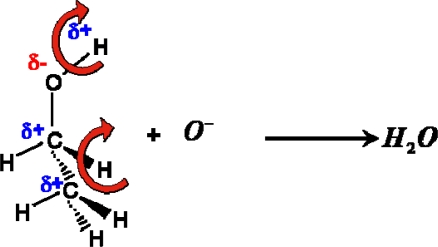


**Figure 10.**
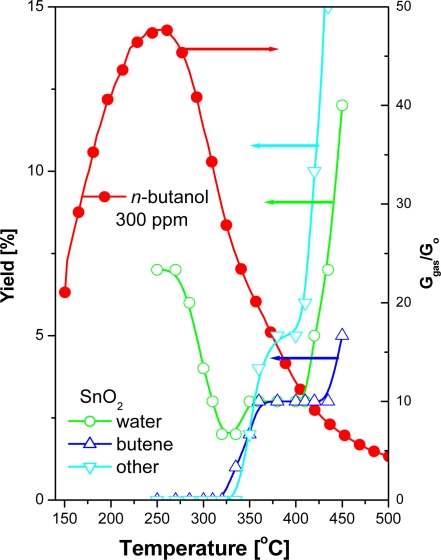
Efficiency of reaction of condensation of *n*-butyl alcohol at the presence SnO_2_ catalyst and sensitivity of sensor based on SnO_2_ in the presence of *n*-butanol.

The authors’ hypothesis has been confirmed by the studies of catalytic activity of tin dioxide for the conversion of *n*-butanol. The studies concerned the efficiency and selectivity of bimolecular condensation reaction of alcohol. The studies showed that in the reaction of bimolecular condensation of *n*-butanol in the presence of tin dioxide at the working temperature of the sensor, water is the main product of the reaction (Figure 10).

The obtained results are important from the point of view of possible modification of the parameters of chemical sensors like sensitivity, working temperature and selectivity. Since the kinetics of the oxidation process of organic compounds at the surface of oxide semiconductors depends on their Lewis acidity and ease of removing of hydrogen atom from the molecule, then it is possible to control the sensitivity, working temperature and selectivity by modification of the Lewis acidity.

## 4. Conclusions

In the work the effect of molecule structure of an organic compound on sensitivity and working temperature of thick-film sensors based on tin dioxide has been evaluated. The studies showed that the electrical performance of resistive sensors depend on acidity of the molecule and ease of removing of hydrogen atoms from alkyl groups. Lewis acidity decreases with the increase in the number of alkyl groups in the molecule whereas the ease of hydrogen removal is reduced when the order of carbon atoms decreases.

Based on the analysis of obtained results and examination of physical-chemical properties of tested organic compounds it has been assumed that the first slowest stage determining kinetics of the oxidation reaction of the compounds and thus determining the performance of the sensor is the process of removal of two hydrogen atoms from the alcohol molecule in the reaction with oxygen chemically adsorbed on the surface of sensing material.

In order to verify the above assumption, catalytic activity of tin dioxide in bimolecular condensation reaction has been assessed. Results of the investigations confirmed the validity of this assumption. As is commonly accepted, during a sensor operation chemical reactions of organic compounds with oxygen chemisorbed at the surface of sensing material take place. From the performed studies it follows that the rate of the stage determining the oxidation process of organic compounds at oxide surfaces of semiconductors depends on Lewis acidity of organic compounds. Thus, by modifying the Lewis acidity of the surface of a sensing material it is possible to affect not only sensitivity and temperature of assaying the compounds but also, what is very important, the selectivity of the sensor.

## Figures and Tables

**Table 1.** Working temperature (T_wr_) and sensitivity (S_600ppm_) of gas sensors in the atmosphere containing different organic compounds.

**Organic compound**	**Chemical formula**	**Number of carbon atoms**	**T_wr_ [°C]**	**Sensitivity S_600ppm_**	**μ [D]**	**ɛ_r_**

*n*-butanol	CH_3_(CH_2_)_3_OH	4	260	73.4	1.66	17.1
2-propanol	CH_3_CHOHCH_3_	3	310	25.4	1.68	18.3
ethanol	CH_3_CH_2_OH	2	330	23.8	1.71	24.3
acetone	CH_3_COCH_3_	3	360	19.8	2.81	20.74
2-methyl-2-propanol	(CH_3_)_3_COH	4	390	16.0	1.65	10.9

* Sensitivity has been evaluated at 600 ppm of examined substance; μ: electrical dipole moment of molecule; ɛ_r_: relative permittivity; T_wr_: working temperature of sensor—temperature at which a sensor exhibits the highest sensitivity for an assumed concentration of tested compounds.
